# 

**Published:** 1897-02

**Authors:** 


### CONTENTS.

ORIGINAL COMMUNICATIONS. PAGE

The Degenerate Jaws and Teeth. By EUGENE TALBOT, M.D., D.D.S. . . . . . 69

Some Thoughts on the Care and Management of the Deciduous Teeth. By S. H. GUILFORD, D.D.S., Ph.D. . . . . . . . . . . . . . . . . . . . . . . . . 85

Annual Address. By JAMES M'MANUS, D.D.S., Hartford, Conn. . . . . . . . 90

On "Five-Minute Papers." By CHARLES M'MANUS, D.D.S. . . . . . . . . . . 96

A Non-Toxic Local Anæsthetic. By W. H. JONES, D.D.S., Fultonville, N. Y. . 98

Nitrate of Silver in Root-Canals. By CHARLES D. CHENEY, Hoboken, N. J. . . 99

ABSTRACTS AND TRANSLATIONS.

Silver and its Salts as Surgical Antiseptics. By DR. B. CREDÉ . . . . . . . . . 101

REPORTS OF SOCIETY MEETINGS.

American Dental Association . . . . . . . . . . . . . . . . . . . . . . 105

The New York Institute of Stomatology . . . . . . . . . . . . . . . . . 119

EDITORIAL.

Principles governing Malleting Force . . . . . . . . . . . . . . . . . . 133

Correction . . . . . . . . . . . . . . . . . . . . . . . . . . . . . . . 137

OBITUARY.

William Newton Morrison, D.D.S. . . . . . . . . . . . . . . . . . . . . 137

DOMESTIC CORRESPONDENCE.

Radiography in Pyorrhœa Alveolaris . . . . . . . . . . . . . . . . . . . 140

